# Predictive Factors of Recurrence for Multifocal Papillary Thyroid Microcarcinoma With Braf^v600e^ Mutation: A Single Center Study of 1,207 Chinese Patients

**DOI:** 10.3389/fendo.2019.00407

**Published:** 2019-06-26

**Authors:** Shuai Xue, Li Zhang, Peisong Wang, Jia Liu, Yue Yin, Meishan Jin, Liang Guo, Yuhua Zhou, Guang Chen

**Affiliations:** ^1^Department of Thyroid Surgery, The 1st Hospital of Jilin University, Changchun, China; ^2^Department of Nephrology, The 1st Hospital of Jilin University, Changchun, China; ^3^Department of Pathology, The 1st Hospital of Jilin University, Changchun, China

**Keywords:** risk stratification, recurrence, papillary thyroid microcarcinoma, multifocality, Braf^V600E^

## Abstract

**Background:** The American Thyroid Association (ATA) guidelines risk stratify Braf^v600e^ mutated multifocal papillary thyroid microcarcinoma (BMPTMC) into different recurrence risk groups by the extent of extrathyroidal extension (ETE). These findings and modifications for BMPTMC need to be verified in additional studies.

**Methods:** A retrospective cohort study was conducted in BMPTMC patients who underwent total thyroidectomy (TT) and central lymph node dissection (CLND) from 2008 to 2013. Overall, 1,207 patients were included, and predictive factors were identified by univariate and multivariate analysis over a mean 7.5-year follow up.

**Results:** BMPTMC with ETE to capsule shows the same recurrence rate (3.8%) with intrathyroidal BMPTMC. Moreover, BMPTMC with ETE only to strap muscle, which belongs to high-risk group according to ATA guideline, shows relatively lower recurrence rate (13.3%) compared with some intermediate risk categories such as cN1 and >5 pN1. Multivariate analysis using a Cox proportional hazards regression model shows that total tumor diameter (TTD) is associated with significantly higher recurrence for BMPTMC with or without other risk factors (Hazard Ratio (HRO) = 9.86 [95%CI 5.35–18.20], *p* = 0.00; HRO = 2.32 [95%CI 1.12–4.85], *p* = 0.02; respectively), while Hashimoto thyroiditis (HT) is found to be protective against the recurrence (HRO = 0.51 [95%CI 0.33–0.79], *p* = 0.00; HRO = 0.47 [95%CI 0.25–0.89], *p* = 0.02; respectively).

**Conclusions:** Taken together, capsular ETE and gross ETE to the strap muscles did not have the expected significant influence on recurrence for Chinese BMPTMC patients who underwent TT and CLND. Rather than the extent of ETE, TTD and the lack of HT were identified as predictors for recurrence among BMPTMC with or without other risk factors (vascular invasion, cN1, pN1>5, pN1>3 cm).

## Introduction

During the past decades, the global incidence of papillary thyroid carcinoma (PTC) has increased substantially. This has been driven largely by the rise in papillary thyroid microcarcinoma (PTMC), which is defined as PTC measuring ≤ 1 cm in greatest dimension (1–3). Although the majority of PTMCs are indolent with a good prognosis, some may have locoregional recurrence, which is still a major concern for clinicians (4, 5). Therefore, many studies have tried to identify several clinical, pathologic and immunohistochemistry predictors of recurrence and/or aggressiveness for PTMC, such as younger age, male sex, multifocality, extrathyroidal extension (ETE), Galectin-3 and Braf mutation, in order to determine an efficacious treatment for different risk groups of PTMC (4, 6–8). However, the findings from the different studies have varied and the conflicting data has contributed to controversial risk stratification (RS) and therapeutic strategies for PTMC.

In 2012, Niemeier et al. developed a molecular-pathological (MP) score including ETE, multifocality, tumor fibrosis, and Braf^v600e^ by comparing 29 aggressive PTMC with 30 non-aggressive tumors (9). They also stratified another 40 PTMCs into low, moderate and high-risk (HR) groups according to the MP score and predicted that the likelihood of lymph node metastases (LNM) or recurrence were 0, 20, and 60%, respectively (9). Based on this study, the American Thyroid Association (ATA) categorized Braf^v600e^ mutated multifocal papillary thyroid microcarcinoma (BMPTMC) with ETE into the intermediate-risk (IR) group of recurrence (10). Meanwhile, intrathyroidal BMPTMC was categorized into the low risk (LR) group because the overall clinical recurrence rate was quite low (1–7%) although 30–73% of cases present with Braf^v600e^ mutation (11, 12). BMPTMC was distinguished from the PTMC cohort and stratified into different recurrence risk groups independently according to the extent of ETE. These findings and modifications for BMPTMC need to be verified in additional studies with larger cohorts.

The aim of this study was to identify clinicopathological features to predict recurrence for BMPTMC by conducting a retrospective analysis of our clinical PTMC cohort, and help physicians to differentiate aggressive BMPTMC, and further guide treatment strategies.

## Materials and Methods

### Patient Selection

**Figure 1:**
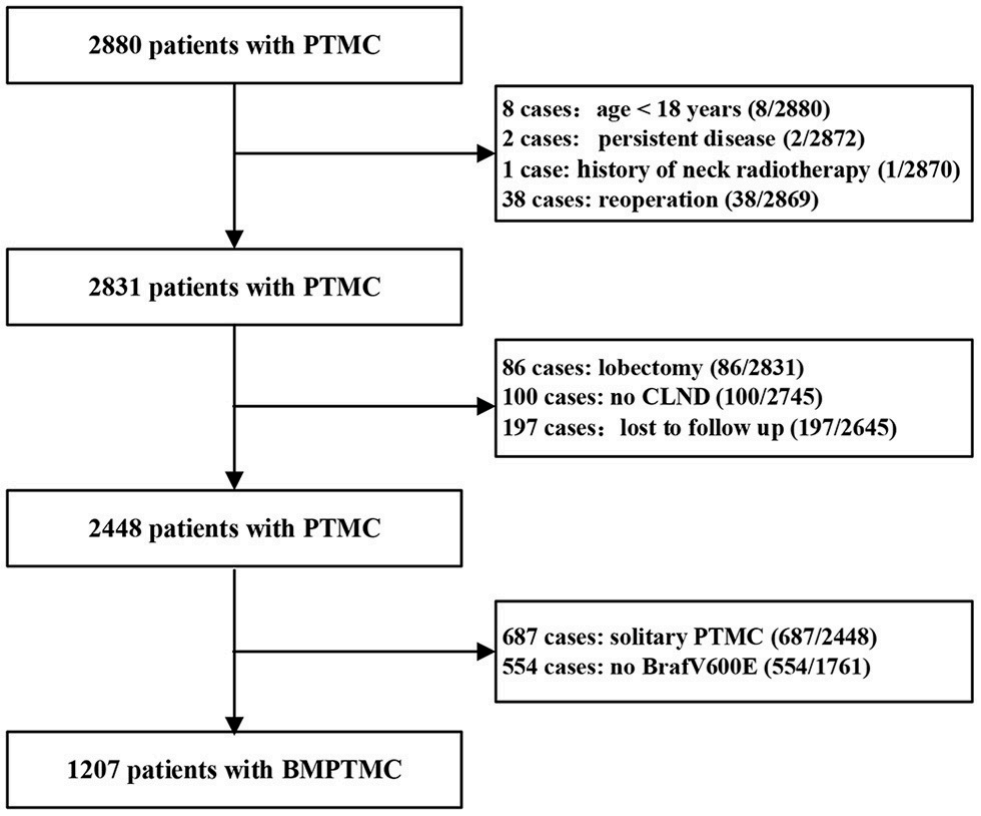
Flowchart showing patient selection.

The Institutional Review Board of the 1st Hospital of the Jilin University approved this study and written informed consent was waived due to the retrospective nature of the study. A total of 2,880 consecutive PTMC patients from January 2008 to January 2013, who underwent initial surgery at the 1st Hospital of Jilin University were retrospectively analyzed. Inclusion criteria for patient selection were as follows: patient information found in a hospital database; total thyroidectomy (TT) with central lymph node dissection (CLND) with or without therapeutic lateral lymph node dissection (LLND) as initial surgery; postoperative pathological diagnosis of multifocal conventional PTMC; molecular analysis of tumor found Braf^v600e^ mutation positive. Exclusion criteria were as follows: aged <18 years; persistent disease; history of neck radiotherapy; history of previous thyroid surgery; lost to follow up. Finally, 1,207 BMPTMC patients were enrolled in our study (Figure 1).

### Diagnosis and Treatment

The majority of PTMC patients were asymptomatic and thyroid carcinomas were identified by routine ultrasound (US) examination, which was conducted to evaluate thyroid tumor and neck lymph nodes by a trained radiologist (Y Yin) and surgeons preoperatively (S Xue, PS Wang, J Liu). If the thyroid nodule was larger than 5 mm and the US was considered suspicious, it was recommended that fine needle aspiration (FNA) be conducted. If the diameter of the thyroid nodule was <5 mm, the physician explained both the risks and benefits of surgery to the patients and a decision was made based on the patients' preference. TT was not recommended for solitary PTMC without other high-risk factors (gross ETE, aggressive histology, vascular invasion, clinical N1). TT and bilateral CLND were routine operations used for patients with bilateral thyroid microcarcinoma. As there is a lack of standard treatment and consensus regarding unilateral multifocal PTMC, patients chose lobectomy (LT) or TT after they were informed about the potential risks and benefits, such as recurrence, surgery complication, radioactive iodine (RAI) ablation, and levothyroxine consumption. CLND was performed as previously described; all lymph nodes and fibro-fatty tissues were removed vertically from the hyoid bone to the thoracic inlet and laterally from the common carotid artery to the midline of the trachea including the lymph nodes posterior to the right laryngeal recurrent nerve (13). Modified LLND including levels II–V with the preservation of the internal jugular vein, spinal accessory nerve, and sternocleidomastoid muscle was only performed for patients with clinical N1, which was diagnosed using US, CT and FNA (13). RAI was recommended to patients who underwent TT in accordance with the published guidelines as previously described (14). However, the decision was made according to both physician and patient preference. TSH-suppressive hormonal therapy was recommended to postoperative patients since 2011 according to established guidelines (13).

### Histopathologic Examination

Histological specimens were examined and reviewed independently by two pathologists (L Guo and YH Zhou). Histopathological characteristics including diameter of all tumors, location of tumors, number of tumor foci, extrathyroidal extension, presence of Hashimoto thyroiditis (HT), and LNM (the number and diameter) were recorded. The concordance rate between the two pathologists for the 1,207 BMPTMC patients included in this study was 98.7%. The very few discordant cases were discussed with the experienced pathologist (MS Jin).

### Follow Up and Recurrence

All cases were followed up with physical examinations, serum unstimulated thyroglobulin (Tg), Tg antibodies, and US at 6- to 12- month intervals. It was recommended that patients who received RAI should receive diagnostic iodine-131 whole body scans. When recurrence was suspected, patients underwent FNA with or without the measurement of washout Tg levels and thyroid CT. In our study, recurrence was defined as the presence of a tumor or metastatic lymph node in a patient who has been considered clinically free of disease at least 6 months after the initial surgery. Overall, 781 patients were followed up in our outpatient facility while 426 patients were followed up by telephone and mail. The follow-up period ranged from 5 to 10 years.

### Molecular Analysis

Braf^V600E^ mutation analysis was performed on paraffin embedded sections of primary tumors obtained after TT. Sections with the largest tumor foci were marked and provided by pathologists. Tumor deparaffinization and DNA extraction were performed using a spinal column procedure (AmoyDx1 FFPE DNA Kit, Amoy Diagnostics, China). The concentration and purity of DNA samples were measured by a NanoDrop ND-1000 spectrophotometer (NanoDrop Technologies, USA). The A260/A280 values between 1.8 and 2.0 and the A260/A230 values >2.0 of extracted DNA were qualified for further use. The DNA samples were stored at −20°C.

The Braf^V600E^ mutation status was detected by real-time PCR using the AmoyDx1 Braf^V600E^ Mutation Detection Kit (Amoy Diagnostics, China) and Bio-Rad CFX96 (Bio-Rad Laboratories, USA) (15, 16). All reactions were done in 40 ul volumes using 5 ul template DNA (15 ng), 35 ul Reaction Mixture and 0.4 ul Taq DNA Polymerase. All needed reagents were included with the kit. PCR cycling conditions were a 5 min hold at 95°C followed by 15 cycles of 95°C for 25 s, 64°C for 20 s, 72°C for 20 s, and 31 cycles of 93°C for 25 s, 60°C for 35 s, 72°C for 20 s, while the fluorescent signals were collected at 60°C according to the manufacturer's instructions. The carboxyfluorescein (FAM) fluorescence signal indicated the mutation status of the sample.

### Statistical Analysis

Nominal variables were described as frequencies and proportions and continuous variables were presented as means and standard deviations (SDs). To identify differences between groups for specific variables, Pearson's chi-square tests were used for nominal variables; continuous variables were turned into nominal variables using cutoffs which were calculated using receiver operating characteristic curve (ROC) analysis. Univariate analysis of risk factors for recurrence using Kaplan–Meier methods and statistically analyzed using the log-rank test. A Cox proportional hazards analysis was used for multivariate analysis. *P* < 0.05 were considered statistically significant (two-sided). SPSS version 22 software (SPSS Inc, Chicago, IL) was used for all statistical analyses.

## Results

### Baseline Characteristics

The baseline clinicopathological and genetic characteristics of the 1,207 patients with BMPTMC were summarized in Table 1. One thousand and forty (86.2%) patients were females and the average age of all patients was 46.9 years. Thirty (2.5%) patients had gross ETE to strap muscles while microscopic ETE was identified among 932 (77.2%) of the BMPTMC patients. Overall, one thousand and ninety-six (90.6%) patients received prophylactic CLND.

**Table 1:** Clinicopathological and molecular genetic characteristics of BMPTMC patients.

**Variables**	***N* = 1207(%)**
**Gender**	
Female	1,040 (86.2)
Male	167 (13.8)
**Age, years**	46.9 ± 12.6
<55	1,055 (87.4)
≥55	152 (12.6)
**Bilateral**	
Yes	948 (78.5)
No	259 (21.5)
**ETE**	
No	245 (20.3)
Microscopic to capsule or soft tissues	932 (77.2)
Gross to strap muscle	30 (2.5)
**Lymphovascular invasion**	
Yes	6 (0.5)
No	1,201 (99.5)
LTD (mm)	6.1 ± 2.1
Number of tumor foci	3 ± 1
TTD (mm)	12 ± 6
**HT**	
Yes	728 (65.2)
No	479 (34.8)
**CLND**	
Prophylactic CLND	1,093 (90.6)
Therapeutic CLND	114 (9.4)
**ATA RS**	
LR	211 (17.5)
IR	964 (79.9)
HR	32 (2.6)
**RAI ablation**	
Yes	405 (33.6)
No	802 (66.4)
**Recurrence**	
Yes	90 (7.5)
No	1,117 (92.5)
Follow up (months)	91 ± 15

*Categorical variables are presented as number (%, percentage). Continuous variables are presented as the average ± standard deviation. BMPTMC, Braf^V600E^ mutated multifocal papillary thyroid microcarcinoma; ETE, extrathyroidal extension; LTD, largest tumor diameter; TTD, total tumor diameter; HT: Hashimoto's thyroiditis; CLND, central lymph node dissection; ATA, American Thyroid Association; RS, risk stratification; LR, low risk; IR, intermediate risk; HR, high risk; RAI, radioactive iodine*.

The average follow up duration was 91 ± 15 months. Recurrence was detected in 90 (7.5%) of the 1,207 BMPTMC patients. The vast majority of recurrence (68/90, 75.6%) was identified in the lateral lymph node (LLN), followed by the central lymph node (CLN) (18/90, 20%), and the operation site (4/90, 4.4%). Moreover, the mean ± SD recurrence time was 85 ± 21 months.

### RS of Recurrence According to the 2015 ATA Guidelines

**Figure 2:**
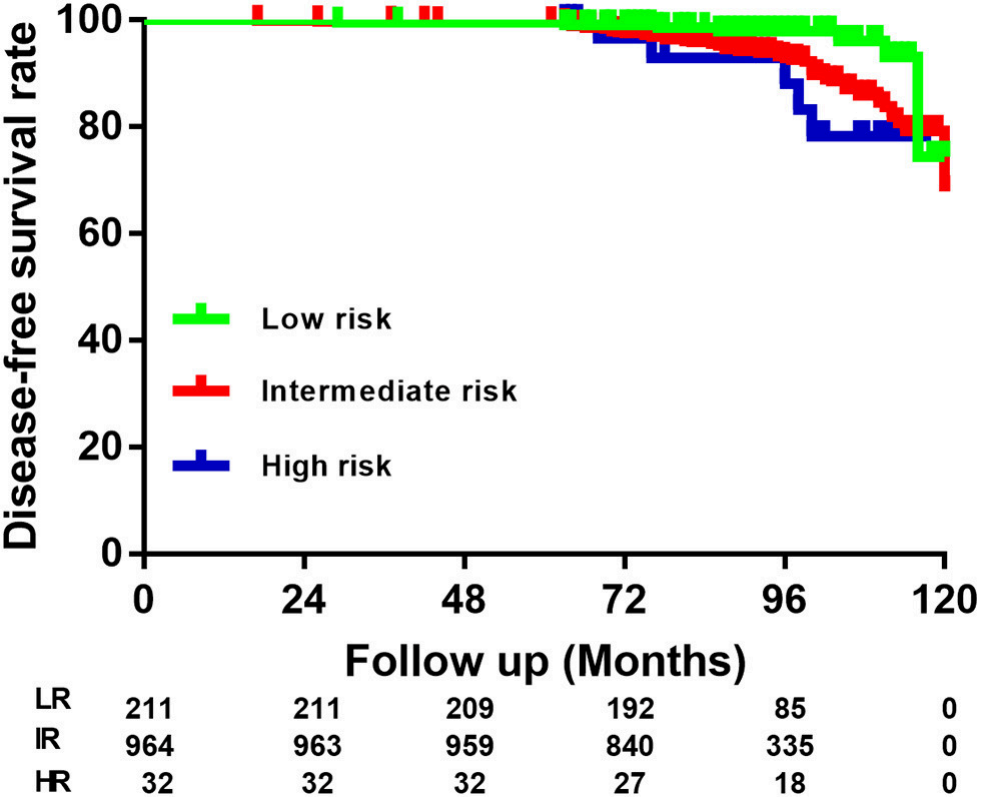
Risk stratification of Braf^v600e^ mutated multifocal papillary thyroid microcarcinoma patients according to the American Thyroid Association guidelines.

The distribution of patients according to the 2015 ATA RS guidelines on recurrence was as follows: LR in 211 (17.5%) patients, IR in 964 (79.9%) patients, and HR in 32 (2.6%) patients. Specific categories of each RS group are shown as Table 2 and Figure 2. It is noteworthy that BMPTMC with ETE to capsule show the same recurrence rate (3.8%) with intrathyroidal BMPTMC. Moreover, BMPTMC with ETE only to strap muscle, which belongs to HR group, show relatively lower recurrence rate (13.3%) compared with some IR categories such as cN1 and >5 pN1.

**Table 2:** Recurrence rates and RS for BMPTMC cohort.

**BMPTMC patients**	**Recurrence/total cases, actual RR%**	**ATA RS**
Intrathyroidal	8/211, 3.8%	LR
ETE to capsule	22/580, 3.8%	IR
ETE to soft tissues	12/183, 6.6%	IR
cN1 or >5 pN1	7/57, 25.9%	IR
Two or more IR factors	36/174, 20.1%	IR
ETE to strap muscle	4/30, 13.3%	HR
pN1 > 3 cm	1/2, 50%	HR

*RS, risk stratification; BMPTMC, Braf^v600e^ mutated multifocal papillary thyroid microcarcinoma; RR, recurrence rate; ATA, American Thyroid Association; ETE, extrathyroidal extension; cN1, clinically N1; pN1, pathologic N1; LR:low risk; IR, intermediate risk; HR, high risk*.

### Risk Factors of Recurrence for 1,207 BMPTMC

Univariate analysis for variables associated with recurrence was performed, including sex, age, bilateral, vascular invasion, extent of ETE, largest tumor diameter (LTD), number of tumor foci, total tumor diameter (TTD), Hashimoto thyroiditis (HT), total number of metastatic CLN (MCLN), diameter of largest MCLN, and RAI ablation. For continuous variables, optimal cutoffs were determined by ROC analysis (Supplementary Table 1). Upon univariate analysis, sex, LTD, TTD, HT, total number of MCLN and the diameter of the largest MCLN were associated with recurrence (*p* = 0.01, 0.04, 0.00, 0.00, 0.00, and 0.00 respectively, Table 3). Multivariate analysis using a Cox proportional hazards regression model showed that TTD >9 mm and total number of MCLN>2 was associated with significantly worse DFS (HRO = 9.86 [95%CI 5.35–18.20], *p* = 0.00; HRO = 4.20 [95%CI 2.47–7.15], *p* = 0.00;), while HT was found to be protective against the recurrence of BMPTMC (HRO = 0.51 [95%CI 0.33–0.79], *p* = 0.00).

**Table 3:** Univariate and multivariate analysis of clinicopathological characteristics for 1207 BMPTMC.

**Variables**	**R(+) *n* = 90**	**R(–) *n* = 1,117**	**Univariate analysis**		**Multivariate analysis**	
			**Hazard ratio (95% CI)**	***P*-value**	**Hazard ratio (95% CI)**	***P*-value**
**Sex**						
Female	85 (94.4)	955 (85.5)	**1 (reference)**	**0.01**	1 (reference)	
Male	5 (0.6)	162 (14.5)	**0.48 (0.27–0.86)**		0.42 (0.17–1.06)	0.07
**Age (years)**						
<55	76 (84.4)	979 (87.6)	1 (reference)		1 (reference)	
≥55	14 (15.6)	138 (12.4)	1.38 (0.73–2.61)	0.32	0.99 (0.53–1.83)	0.96
**Bilateral**						
Yes	66 (73.3)	882 (79.0)	0.85 (0.52–1.39)		0.89 (0.53–1.49)	
No	24 (26.7)	235 (21.0)	1 (reference)	0.52	1 (reference)	0.65
**Vascular invasion**						
Yes	1 (1.1)	5 (0.4)	1.80 (0.15–21.55)		2.05 (0.28–15.18)	
No	89 (98.9)	1,112 (99.6)	1 (reference)	0.64	1 (reference)	0.48
**ETE**						
No	17 (18.9)	228 (20.4)	1 (reference)		1 (reference)	
Microscopic to capsule or soft tissues	69 (76.7)	863 (77.3)	1.17 (0.71–1.93)		0.85 (0.47–1.53)	0.59
Gross to strap muscle	4 (4.4)	26 (2.3)	2.00 (0.58–6.89)	0.38	1.40 (0.49–4.03)	0.53
**LTD (mm)**						
≤ 5	28 (31.1)	461 (41.3)	**1 (reference)**		1 (reference)	
>5	62 (68.9)	656 (58.7)	**1.55 (1.01–2.36)**	**0.04**	1.54 (1.31–1.97)	0.05
**Number of tumor foci**						
≤ 3	55 (61.1)	748 (67.0)	1 (reference)		1 (reference)	
>3	35 (38.9)	369 (33.0)	1.41 (0.91–2.19)	0.12	1.07 (0.52–2.78)	0.06
**TTD (mm)**						
≤ 9	22 (24.4)	389 (34.8)	**1 (reference)**		**1 (reference)**	
>9	68 (75.6)	728 (65.2)	**15.71 (8.63–28.60)**	**0.00**	**9.86 (5.35–18.20)**	**0.00**
**HT**						
Yes	43 (47.8)	685 (61.3)	**0.56 (0.36–0.85)**		**0.51 (0.33–0.79)**	
No	47 (52.2)	432 (38.7)	**1 (reference)**	**0.00**	**1 (reference)**	**0.00**
**Total number of MCLN**						
≤ 2	40 (44.4)	925 (82.8)	**1 (reference)**		**1 (reference)**	
>2	50 (55.6)	192 (17.2)	**11.59 (6.81–19.74)**	**0.00**	**4.20 (2.47–7.15)**	**0.00**
**Diameter of largest MCLN (mm)**						
≤ 5	43 (47.8)	848 (75.9)	**1 (reference)**		1 (reference)	
>5	47 (52.2)	269 (24.1)	**5.18 (3.17–8.47)**	**0.00**	1.49 (0.87–2.55)	0.15
**RAI**						
Yes	19 (21.1)	380 (34.0)	0.83 (0.53–1.31)		1.52 (0.91–2.54)	
No	71 (78.9)	737(66.0)	1 (reference)	0.43	1 (reference)	0.11

*BMPTMC, Braf^V600E^ mutated multifocal papillary thyroid microcarcinoma; R, recurrence; ETE, extrathyroidal extension; LTD, largest tumor diameter; TTD, total tumor diameter; HT, Hashimoto's thyroiditis; MCLN, metastatic central lymph node; MLLN, metastatic lateral lymph node; RAI, radioactive iodine. Bold values indicate the statistically significant*.

### Risk Factors for Recurrence for 1004 BMPTMC Without Other Risk Factors

BMPTMC patients with other risk factors (vascular invasion, cN1, pN1>5, pN1>3 cm) will be risk stratified to IR or HR group only according to these risk factors. For 1004 BMPTMC without other risk factors, univariate analysis for variables associated with recurrence was performed, including sex, age, bilateral, extent of ETE, LTD, number of tumor foci, TTD, HT, total number of MCLN, diameter of largest MCLN, and RAI ablation. For continuous variables, optimal cutoffs were determined by ROC analysis (Supplementary Table 2). Upon univariate analysis, the number of tumor foci, TTD, HT, and the diameter of the largest metastatic CLN were associated with DFS (*p* = 0.01, 0.00, 0.00, and 0.01 respectively, Table 4). Multivariate analysis using a Cox proportional hazards regression model showed that TTD >15 mm was associated with significantly higher recurrence (HRO = 2.32 [95%CI 1.12–4.85], *p* = 0.02), while HT was found to be protective against the recurrence of BMPTMC (HRO = 0.47 [95%CI 0.25–0.89], *p* = 0.02).

**Table 4:** Univariate and multivariate analysis of clinicopathological characteristics for BMPTMC without other risk factors (vascular invasion, cN1, pN1 > 5, pN1 > 3 cm).

**Variables**	**R(+) *n* = 46**	**R(–) *n* = 958**	**Univariate analysis**		**Multivariate analysis**	
			**Hazard ratio (95% CI)**	***P*-value**	**Hazard ratio (95% CI)**	***P*-value**
**Sex**						
Female	43 (93.5)	805 (84.0)	1 (reference)		1 (reference)	
Male	3 (6.5)	153 (16.0)	0.49 (0.22–1.09)	0.08	0.41 (0.13–1.32)	0.13
**Age (years)**						
<55	42 (91.3)	857 (89.5)	1 (reference)		1 (reference)	
≥55	4 (8.7)	101 (10.5)	0.85 (0.32–2.21)	0.74	1.33 (0.45–3.92)	0.61
**Bilateral**						
Yes	35 (76.1)	765 (79.9)	0.99 (0.50–1.96)		0.57 (0.26–1.21)	
No	11 (23.9)	193 (20.1)	1 (reference)	0.98	1 (reference)	0.14
**ETE**						
No	8 (17.4)	203 (21.2)	1 (reference)		1 (reference)	
Microscopic to capsule or soft tissues	34 (73.9)	729 (76.1)	1.32 (0.66–2.64)	0.19	1.36 (0.60–3.05)	0.46
Gross to strap muscle	4 (8.7)	26 (2.7)	2.92 (0.60–14.25)		1.92 (0.53–6.95)	0.32
**LTD (mm)**						
≤ 7	35 (76.1)	704 (73.5)	1 (reference)		1 (reference)	
>7	11 (23.9)	254 (26.5)	0.88 (0.45–1.69)	0.69	0.54 (0.25–1.14)	0.11
**Number of tumor foci**						
= 2	10 (21.7)	372 (38.8)	**1 (reference)**		1 (reference)	
>2	36 (78.3)	586 (61.2)	**2.13 (1.18–3.86)**	**0.01**	2.07 (0.90–4.78)	0.09
**TTD (mm)**						
≤ 15	27 (58.7)	746 (77.9)	**1 (reference)**		**1 (reference)**	
>15	19 (41.3)	212 (22.1)	**3.41 (1.67–6.96)**	**0.00**	**2.32 (1.12–4.85)**	**0.02**
**HT**						
Yes	19 (41.3)	589 (61.5)	**0.42 (0.23–0.76)**		**0.47 (0.25–0.89)**	
No	27 (58.7)	369 (38.5)	**1 (reference)**	**0.00**	**1 (reference)**	**0.02**
**Total number of MCLN**						
≤ 4	40 (87.0)	908 (94.8)	1 (reference)		1 (reference)	
>4	6 (13.0)	50 (5.2)	2.20 (0.73–6.63)	0.16	1.17 (0.45–3.04)	0.75
**Diameter of largest MCLN (mm)**						
≤ 6	38 (82.6)	867 (90.5)	**1 (reference)**		1 (reference)	
>6	8 (17.4)	91 (9.5)	**3.86 (1.30–11.39)**	**0.01**	2.17 (0.96–4.88)	0.06
**RAI**						
Yes	10 (21.7)	326 (34.0)	0.69 (0.36–1.31)		0.68 (0.33–1.41)	
No	36 (78.3)	632 (66.0)	1 (reference)	0.25	1 (reference)	0.30

*BMPTMC, Braf^V600E^ mutated multifocal papillary thyroid microcarcinoma; R, recurrence; ETE, extrathyroidal extension; LTD, largest tumor diameter; TTD, total tumor diameter; HT, Hashimoto's thyroiditis; MCLN, metastatic central lymph node; MLLN, metastatic lateral lymph node; RAI, radioactive iodine. Bold values indicate the statistically significant*.

## Discussion

To our knowledge, this is the first paper to investigate predictive factors of recurrence for BMPTMC who underwent TT and CLND. In our BMPTMC cohort with aggressive treatment, 90 of the 1,207 (7.5%) patients presented with recurrence. Overall, 763 BMPTMC patients were categorized into the IR recurrence group only due to the concurrence with microscopic ETE. However, only 34 (34/763, 4.5%) patients were diagnosed with recurrence; These findings were not in agreement with the expected recurrence rates (10–40%) for IR (10). BMPTMC with ETE was classified into IR as a result of the findings from Niemeier et al.'s study in 2012 (9). Different characteristics of study subjects, treatment strategy and follow-up time and methods may be the potential reasons for this discrepancy.

More interestingly, only four of 30 (13.3%) BMPTMC patients with gross ETE to strap muscle were diagnosed with recurrence. SY Park et al also investigated 260 differentiated thyroid carcinoma (DTC) patients who underwent TT and RAI with mean follow up period of 10.6 years; they found 38 (10.8%) recurrent cases (17). It is well-known that the degree of ETE in PTC plays important roles in disease-specific survival (DSS) and disease-free survival (DFS) (18). However, it was found that gross ETE to strap muscle had little effect on DSS and DFS which has been already verified by mounting evidence (17, 19, 20). It has been reported that DTC patients with strap muscular gross ETE had the same DFS as those with microscopic ETE (20). Shaha AR also suggested that detailed distinguishing of the degree of gross ETE should be performed (19). Patients with anterior extrathyroidal extension involving the strap muscle had a relatively good prognosis compared with posterior gross ETE to recurrent laryngeal nerve. This is due to the fact that gross ETE to strap muscle can be easily resected with negative margins (21).

Additionally, there has been considerable debate over the years regarding the role of tumoral multifocality in recurrence, persistence, metastasis, and prognosis (22). Conflicting data may be the result of different clinicopathological features regarding multifocal thyroid cancer cohorts, such as the number, size, and location of tumor foci. The impact of additional small tumor foci on aggressiveness is also controversial. The only evaluation for the largest tumor foci may lead to underestimation of tumor behavior for multifocal thyroid cancer (22). TTD is believed to be a better parameter to represent multifocal thyroid cancer aggressiveness (23, 24). TTD>1 cm is associated with more LNM, capsule invasion, and worse DFS than TTD ≤ 1 cm (25). Compared with unifocal PTC >1 cm, TTD >1 cm also confers a similar risk of aggressive tumor behaviors like ETE and LNM (26, 27). Based on these evidence, AA Tam et al recommended that T1a multifocal PTMC (TTD 1–2 cm) be reclassified into T1b (28). In our study, we also identified TTD as risk factor of recurrence among BMPTMC patients with or without other risk factors (vascular invasion, cN1, pN1>5, pN1>3 cm). Recently, AA Tam et al also found that a lower tumor diameter ratio (LTD/TTD) can predict capsular invasion, ETE, and LNM among multifocal thyroid cancer accurately; this, however, requires validation in other studies (22).

Moreover, multiple studies and meta-analyses have proven that PTC with HT had more favorable clinicopathological features and better prognosis than those without HT. This is in agreement with our results (29–31). It has been reported that HT is a potential protective factor for PTC, regardless of the BrafV600E mutation (32–34). However, the underlying molecular mechanism of the association between HT and PTC is still unknown. The theory of “tumor defense-induced autoimmunity” may explain the coexistence of HT and PTC (35). The immune cell infiltration into the thyroid is believed to be an ongoing antitumor immune response, which leads to a better prognosis among PTC patients (35).

There are huge concerns for aggressive treatment of PTMC in our cohort, such as FNA for >5 mm PTMC, routine prophylactic CLND and more RAI ablation. These therapeutic strategies are different with current ATA or BTA guidelines. However, we can explain this as follows: (1) Patients enrolled in this study are diagnosed and treated from 2008 to 2013. At that time, treatments of PTMC were controversial and no reliable guideline has been published. (2) Some treatments of PTMC like FNA for >5 mm suspicious and routine prophylactic CLND are still consistent with guideline of Chinese Anti-Cancer Association (36). (3) Whether aggressive treatment of PTMC will lead to lower recurrence and better prognosis is still debatable (37–39). Accordingly, we don't think these differences of treatments for PTMC will impact the results remarkably in our study.

Several limitations in the study must be noted. First, it was a retrospective single-center study at a tertiary medical center. The potential selection bias may limit the generalizability of these findings on a broader scale. Further prospective studies are required to investigate this. Second, the average follow-up time was 7.4 years (89 months), which may be shorter than recurrence time for PTMC. Third, in addition to the number and diameter of metastatic lymph nodes, extranodal extension has been identified as prognostic factor for thyroid cancer (40). However, extranodal extension was not mandated in the pathologic report before 2013 in our hospital, which may generate some bias in our study. Fourth, the prevalence and potential influence of prognosis by Braf^v600e^ mutation were different between races (41). Studies from East Asian counties such as Japan and South Korea reported more than 60% Braf^v600e^ mutation rate among PTC patients, while only 30–50% in American and Europe (41–45). In our study, 69% (1,207/1,761) multifocal PTMC patients had Braf^v600e^ mutation, which indicated the results of this study should be interpreted with caution and need to be validated by BMPTMC cohort from other countries. Fifth, 426 patients in our study were followed up by telephone and mail. The vast majority of rural patients were followed up postoperatively in primary or secondary referral hospital near their home at 6–12 months interval. We followed up them by telephone and mail every 6 month and required recent thyroid function test (including Tg and Tg antibody) updated. When recurrence was suspected, patients were recommended return our hospital to diagnosis. Based on some probably inaccurate information provided by patients through telephone and mail, a small amount of recurrent cases may be not found promptly. However, all recurrent cases were re-evaluated and diagnosed in our hospital. Finally, since only conventional PTC patients were enrolled in this study, the results of this study cannot be applied to other variants of BMPTMC.

## Conclusion

In conclusion, capsular ETE and gross ETE to the strap muscles did not have the expected significant influence on recurrence for Chinese BMPTMC patients who underwent TT and CLND. Rather than the extent of ETE, larger TTD and the lack of HT were identified as predictors for recurrence among those with BMPTMC with or without other risk factors (vascular invasion, cN1, pN1>5, pN1>3 cm), although future prospective studies with more BMPTMC patients and longer follow up are warranted.

## Data Availability

The datasets generated for this study are available on request to the corresponding author.

## Author Contributions

All authors listed have made a substantial, direct and intellectual contribution to the work, and approved it for publication.

### Conflict of Interest Statement

The authors declare that the research was conducted in the absence of any commercial or financial relationships that could be construed as a potential conflict of interest.

## References

[1] Lim H Devesa SS Sosa JA Check D Kitahara CM. Trends in thyroid cancer incidence and mortality in the United States, 1974-2013. JAMA. (2017) 317:1338–48. 10.1001/jama.2017.2719 28362912 PMC8216772

[2] Kitahara CM Sosa JA. The changing incidence of thyroid cancer. Nat Rev Endocrinol. (2016) 12:646–53. 10.1038/nrendo.2016.110 27418023 PMC10311569

[3] Hedinger C Williams ED Sobin LH. The WHO histological classification of thyroid tumors: a commentary on the second edition. Cancer. (1989) 63:908–11. 10.1002/1097-0142(19890301)63:5<908::AID-CNCR2820630520>3.0.CO;2-I 2914297

[4] Siddiqui S White MG Antic T Grogan RH Angelos P Kaplan EL. Clinical and pathologic predictors of lymph node metastasis and recurrence in papillary thyroid microcarcinoma. Thyroid. (2016) 26:807–15. 10.1089/thy.2015.0429 27117842

[5] Pisanu A Saba A Podda M Reccia I Uccheddu A. Nodal metastasis and recurrence in papillary thyroid microcarcinoma. Endocrine. (2015) 48:575–81. 10.1007/s12020-014-0350-7 25007850

[6] Chen Y Sadow PM Suh H Lee KE Choi JY Suh YJ. BRAF(V600E) is correlated with recurrence of papillary thyroid microcarcinoma: a systematic review, multi-institutional primary data analysis, and meta-analysis. Thyroid. (2016) 26:248–55. 10.1089/thy.2015.0391 26671072

[7] Kim TY Hong SJ Kim JM Kim WG Gong G Ryu JS. Prognostic parameters for recurrence of papillary thyroid microcarcinoma. BMC Cancer. (2008) 8:296. 10.1186/1471-2407-8-296 18851763 PMC2576338

[8] Trimboli P Virili C Romanelli F Crescenzi A Giovanella L. Galectin-3 performance in histologic a cytologic assessment of thyroid nodules: a systematic review and meta-analysis. Int J Mol Sci. (2017) 18:1756. 10.3390/ijms18081756 28800068 PMC5578146

[9] Niemeier LA Kuffner Akatsu H Song C Carty SE Hodak SP Yip L. A combined molecular-pathologic score improves risk stratification of thyroid papillary microcarcinoma. Cancer. (2012) 118:2069–77. 10.1002/cncr.26425 21882177 PMC3229649

[10] Haugen BR Alexander EK Bible KC Doherty GM Mandel SJ Nikiforov YE. 2015 American thyroid association management guidelines for adult patients with thyroid nodules and differentiated thyroid cancer: the American thyroid association guidelines task force on thyroid nodules and differentiated thyroid cancer. Thyroid. (2016) 26:1–133. 10.1089/thy.2015.0020 26462967 PMC4739132

[11] Xing M. BRAF mutation in papillary thyroid cancer: pathogenic role, molecular bases, and clinical implications. Endocr Rev. (2007) 28:742–62. 10.1210/er.2007-0007 17940185

[12] Elisei R Viola D Torregrossa L Giannini R Romei C Ugolini C. The BRAF(V600E) mutation is an independent, poor prognostic factor for the outcome of patients with low-risk intrathyroid papillary thyroid carcinoma: single-institution results from a large cohort study. J Clin Endocrinol Metab. (2012) 97:4390–8. 10.1210/jc.2012-1775 23066120

[13] Xue S Wang P Liu J Chen G. Radioactive iodine ablation decrease recurrences in papillary thyroid microcarcinoma with lateral lymph node metastasis in Chinese patients. World J Surg. (2017) 41:3139–46. 10.1007/s00268-017-4134-0 28741199 PMC5680383

[14] Cooper DS Doherty GM Haugen BR Kloos RT Lee SL. Revised American Thyroid Association management guidelines for patients with thyroid nodules and differentiated thyroid cancer. Thyroid. (2009) 19:1167–214. 10.1089/thy.2009.0110 19860577

[15] Xu X Ma X Zhang X Cao G Tang Y Deng X. Detection of BRAF V600E mutation in fine-needle aspiration fluid of papillary thyroid carcinoma by droplet digital PCR. Clin Chim Acta. (2019) 491:91–6. 10.1016/j.cca.2019.01.017 30682328

[16] Shi C Guo Y Lv Y Nanding A Shi T Qin H. Clinicopathological features and prognosis of papillary thyroid microcarcinoma for surgery and relationships with the BRAFV600E mutational status and expression of angiogenic factors. PLoS ONE. (2016) 11:e0167414. 10.1371/journal.pone.0167414 27936049 PMC5147869

[17] Park SY Kim HI Choi JY Choe JH Kim JH Kim JS. Low versus high activity radioiodine remnant ablation for differentiated thyroid carcinoma with gross extrathyroidal extension invading only strap muscles. Oral Oncol. (2018) 84:41–5. 10.1016/j.oraloncology.2018.07.002 30115474

[18] Radowsky JS Howard RS Burch HB Stojadinovic A. Impact of degree of extrathyroidal extension of disease on papillary thyroid cancer outcome. Thyroid. (2014) 24:241–4. 10.1089/thy.2012.0567 23713855

[19] Shaha AR. Extrathyroidal extension-what does it mean. Oral Oncol. (2017) 68:50–2. 10.1016/j.oraloncology.2017.03.008 28438293

[20] Amit M Boonsripitayanon M Goepfert RP Tam S Busaidy NL Cabanillas ME. Extrathyroidal extension: does strap muscle invasion alone influence recurrence and survival in patients with differentiated thyroid cancer? Ann Surg Oncol. (2018) 25:3380–8. 10.1245/s10434-018-6563-x 30022274

[21] Song E Lee YM Oh HS Jeon MJ Song DE Kim TY. A relook at the T stage of differentiated thyroid carcinoma with a focus on gross extrathyroidal extension. Thyroid. (2019) 29:202–8. 10.1089/thy.2018.0300 30358515

[22] Tam AA Ozdemir D Cuhaci N Baser H Dirikoc A Aydin C. Can ratio of the biggest tumor diameter to total tumor diameter be a new parameter in the differential diagnosis of agressive and favorable multifocal papillary thyroid microcarcinoma? Oral Oncol. (2017) 65:1–7. 10.1016/j.oraloncology.2016.12.004 28109462

[23] Buffet C Golmard JL Hoang C Tresallet C Du Pasquier Fediaevsky L Fierrard H. Scoring system for predicting recurrences in patients with papillary thyroid microcarcinoma. Eur J Endocrinol. (2012) 167:267–75. 10.1530/EJE-12-0105 22648965

[24] Pyo JS Sohn JH Kang G Kim DH Yun J. Total surface area is useful for differentiating between aggressive and favorable multifocal papillary thyroid carcinomas. Yonsei Med J. (2015) 56:355–61. 10.3349/ymj.2015.56.2.355 25683981 PMC4329344

[25] Liu C Wang S Zeng W Guo Y Liu Z Huang T. Total tumour diameter is superior to unifocal diameter as a predictor of papillary thyroid microcarcinoma prognosis. Sci Rep. (2017) 7:1846. 10.1038/s41598-017-02165-6 28500312 PMC5431972

[26] Zhao Q Ming J Liu C Shi L Xu X Nie X. Multifocality and total tumor diameter predict central neck lymph node metastases in papillary thyroid microcarcinoma. Ann Surg Oncol. (2013) 20:746–52. 10.1245/s10434-012-2654-2 22972508

[27] Tam AA Ozdemir D Cuhaci N Baser H Aydin C Yazgan AK. Association of multifocality, tumor number, and total tumor diameter with clinicopathological features in papillary thyroid cancer. Endocrine. (2016) 53:774–83. 10.1007/s12020-016-0955-0 27090526

[28] Tam AA Ozdemir D Ogmen BE Faki S Dumlu EG Yazgan AK. Should multifocal papillary thyroid carcinomas classified as T1a with a tumor diameter sum of 1 to 2 centimeters be reclassified as T1b? Endocr Pract. (2017) 23:526–35. 10.4158/EP161488.OR 28156153

[29] Kim SK Woo JW Lee JH Park I Choe JH Kim JH. Chronic lymphocytic thyroiditis and BRAF V600E in papillary thyroid carcinoma. Endocr Relat Cancer. (2016) 23:27–34. 10.1530/ERC-15-0408 26598713

[30] Liang J Zeng W Fang F Yu T Zhao Y Fan X. Clinical analysis of Hashimoto thyroiditis coexistent with papillary thyroid cancer in 1392 patients. Acta Otorhinolaryngol. (2017) 37:393–400. 10.14639/0392-100X-1709 29165434 PMC5720867

[31] Moon S Chung HS Yu JM Yoo HJ Park JH Kim DS. Associations between hashimoto thyroiditis and clinical outcomes of papillary thyroid cancer: a meta-analysis of observational studies. Endocrinol Metab. (2018) 33:473–84. 10.3803/EnM.2018.33.4.473 30513562 PMC6279904

[32] Kim SJ Myong JP Jee HG Chai YJ Choi JY Min HS. Combined effect of Hashimoto's thyroiditis and BRAF(V600E) mutation status on aggressiveness in papillary thyroid cancer. Head Neck. (2016) 38:95–101. 10.1002/hed.23854 25213729

[33] Kwak HY Chae BJ Eom YH Hong YR Seo JB Lee SH Does papillary thyroid carcinoma have a better prognosis with or without Hashimoto thyroiditis? Int J Clin Oncol. (2015) 20:463–73. 10.1007/s10147-014-0754-7 25312294

[34] Zeng RC Jin LP Chen ED Dong SY Cai YF Huang GL. Potential relationship between Hashimoto's thyroiditis and BRAF(V600E) mutation status in papillary thyroid cancer. Head Neck. (2016) 38(Suppl. 1):E1019–25. 10.1002/hed.24149 26041461

[35] Ehlers M Schott M. Hashimoto's thyroiditis and papillary thyroid cancer: are they immunologically linked? Trends Endocrinol Metab. (2014) 25:656–64. 10.1016/j.tem.2014.09.001 25306886

[36] Gao M Ge M Ji Q Cheng R Lu H Guan H. 2016 Chinese expert consensus and guidelines for the diagnosis and treatment of papillary thyroid microcarcinoma. Cancer Biol Med. (2017) 14:203–11. 10.20892/j.issn.2095-3941.2017.0051 28948061 PMC5570598

[37] So YK Seo MY Son YI. Prophylactic central lymph node dissection for clinically node-negative papillary thyroid microcarcinoma: influence on serum thyroglobulin level, recurrence rate, and postoperative complications. Surgery. (2012) 151:192–8. 10.1016/j.surg.2011.02.004 21497873

[38] Hu G Zhu W Yang W Wang H Shen L Zhang H. The effectiveness of radioactive iodine remnant ablation for papillary thyroid microcarcinoma: a systematic review and meta-analysis. World J Surg. (2016) 40:100–9. 10.1007/s00268-015-3346-4 26578322

[39] Kim HJ Kim NK Choi JH Kim SW Jin SM Suh S Radioactive iodine ablation does not prevent recurrences in patients with papillary thyroid microcarcinoma. Clin Endocrinol. (2013) 78:614–20. 10.1111/cen.12034 22957654

[40] Rowe ME Ozbek U Machado RA Yue LE Hernandez-Prera JC Valentino A The prevalence of extranodal extension in papillary thyroid cancer based on the size of the metastatic node: adverse histologic features are not limited to larger lymph nodes. Endocr Pathol. (2018) 29:80–5. 10.1007/s12022-018-9518-7 29396810

[41] Xing M Alzahrani AS Carson KA Viola D Elisei R. Association between BRAF V600E mutation and mortality in patients with papillary thyroid cancer. JAMA. (2013) 309:1493–501. 10.1001/jama.2013.3190 23571588 PMC3791140

[42] Nam JK Jung CK Song BJ Lim DJ Chae BJ Lee NS. Is the BRAF(V600E) mutation useful as a predictor of preoperative risk in papillary thyroid cancer? Am J Surg. (2012) 203:436–41. 10.1016/j.amjsurg.2011.02.013 21803329

[43] Oishi N Kondo T Nakazawa T Mochizuki K Inoue T Kasai K. Frequent BRAF (V600E) and absence of TERT promoter mutations characterize sporadic pediatric papillary thyroid carcinomas in Japan. Endocr Pathol. (2017) 28:103–11. 10.1007/s12022-017-9470-y 28176151

[44] Park YJ Kim YA Lee YJ Kim SH Park SY Kim KW. Papillary microcarcinoma in comparison with larger papillary thyroid carcinoma in BRAF(V600E) mutation, clinicopathological features, and immunohistochemical findings. Head Neck. (2010) 32:38–45. 10.1002/hed.21142 19475551

[45] Elisei R Ugolini C Viola D Lupi C Biagini A Giannini R. BRAF(V600E) mutation and outcome of patients with papillary thyroid carcinoma: a 15-year median follow-up study. J Clin Endocrinol Metab. (2008) 93:3943–9. 10.1210/jc.2008-0607 18682506

