# Supplementary material for: Predictive Factors of Recurrence for Multifocal Papillary Thyroid Microcarcinoma With Brafv600e Mutation: A Single Center Study of 1,207 Chinese Patients
Source: Front Endocrinol (Lausanne). 2019 Jun 26;10:407. doi: 10.3389/fendo.2019.00407 (PMC6607364; doi:10.3389/fendo.2019.00407)
Supplement: Supplementary file 1 [file Table_1.doc]

**Supplementary Table 1: ROC analysis for optimal cutoff of continuous variables**

| Variables | AUC (95% CI) | P value | Youden Index | Cutoff |
| --- | --- | --- | --- | --- |
| LTD (mm) | 0.56(0.53-0.59) | 0.04 | 0.10 | >5 |
| Number of tumor foci | 0.54(0.51-0.56) | 0.24 | 0.06 | >3 |
| TTD (mm) | 0.57(0.54-0.60) | 0.02 | 0.10 | >9 |
| Total number of MCLN | 0.69(0.66-0.72) | 0.00 | 0.38 | >2 |
| Diameter of largest MCLN (mm) | 0.65(0.62-0.68) | 0.00 | 0.28 | >5 |

ROC: receiver operating characteristic curve; AUC: area under curve; LTD: largest tumor diameter; TTD: total tumor diameter; MCLN: metastatic central lymph node;
