# Supplementary material for: Predictive Factors of Recurrence for Multifocal Papillary Thyroid Microcarcinoma With Brafv600e Mutation: A Single Center Study of 1,207 Chinese Patients
Source: Front Endocrinol (Lausanne). 2019 Jun 26;10:407. doi: 10.3389/fendo.2019.00407 (PMC6607364; doi:10.3389/fendo.2019.00407)
Supplement: Supplementary file 2 [file Table_2.doc]

**Supplementary Table 2: ROC analysis for optimal cutoff of continuous variables**

| Variables | AUC (95% CI) | P value | Youden Index | Cutoff |
| --- | --- | --- | --- | --- |
| LTD (mm) | 0.50(0.47-0.53) | 0.95 | 0.03 | >7 |
| Number of tumor foci | 0.59(0.56-0.62) | 0.02 | 0.17 | >2 |
| TTD (mm) | 0.58(0.54-0.61) | 0.10 | 0.15 | >15 |
| Total number of MCLN | 0.52(0.49-0.55) | 0.62 | 0.12 | >4 |
| Diameter of largest MCLN (mm) | 0.51(0.48-0.54) | 0.79 | 0.08 | >6 |

ROC: receiver operating characteristic curve; AUC: area under curve; LTD: largest tumor diameter; TTD: total tumor diameter; MCLN: metastatic central lymph node;
